# Mechanistic Insights into Cytokine Antagonist-Drug Interactions: A Physiologically Based Pharmacokinetic Modelling Approach with Tocilizumab as a Case Study

**DOI:** 10.3390/pharmaceutics17070896

**Published:** 2025-07-10

**Authors:** Xian Pan, Cong Liu, Felix Stader, Abdallah Derbalah, Masoud Jamei, Iain Gardner

**Affiliations:** Certara Predictive Technologies Division, Certara UK Limited, Sheffield S1 2BJ, UK

**Keywords:** tocilizumab, cytokine antagonist, therapeutic protein-drug interaction, interleukine-6, PBPK modelling

## Abstract

**Background:** Understanding interactions between cytokine antagonists and drugs is essential for effective medication management in inflammatory conditions. Recent regulatory authority guidelines emphasise a systematic, risk-based approach to evaluating these interactions, underscoring the need for mechanistic insight. Proinflammatory cytokines, such as interleukin-6 (IL-6), modulate cytochrome P450 (CYP) enzymes, reducing the metabolism of CYP substrates. Cytokine antagonists (such as IL-6 receptor antagonists) can counteract this effect, restoring CYP activity and increasing drug clearance. However, quantitative prediction of cytokine-mediated drug interactions remains challenging, as existing models often lack the mechanistic detail needed to capture the dynamic relationship between cytokine signalling, receptor engagement, and downstream modulation of drug metabolism. **Methods:** A physiologically based pharmacokinetic (PBPK) framework incorporating cytokine–receptor binding, subsequent downregulation of CYP expression, and blockade of the cytokine signalling by a therapeutic protein antagonist was developed to simulate and investigate cytokine antagonist-drug interactions. Tocilizumab, a humanised IL-6 receptor antagonist used to treat several inflammatory conditions associated with elevated IL-6 levels, was selected as a model drug to demonstrate the utility of the framework. **Results:** The developed PBPK model accurately predicted the pharmacokinetics profiles of tocilizumab and captured clinically observed dynamic changes in simvastatin exposure before and after tocilizumab treatment in rheumatoid arthritis (RA) patients. Simulated IL-6 dynamics aligned with observed clinical profiles, showing transient elevation following receptor blockade and associated restoration of CYP3A4 activity. Prospective simulations with commonly co-administered CYP substrates (celecoxib, chloroquine, cyclosporine, ibuprofen, prednisone, simvastatin, and theophylline) in RA patients revealed dose regimen- and drug-dependent differences in interaction magnitude. **Conclusions:** This study demonstrated the utility of PBPK models in providing a mechanistic understanding of cytokine antagonist-drug interactions, supporting enhanced therapeutic decision-making and optimising patient care in inflammatory conditions.

## 1. Introduction

Therapeutic protein-drug interactions (TP-DIs), particularly those related to cytokine modulators, have emerged as a critical area of investigation for ensuring optimal drug efficacy and safety in patients with inflammatory conditions. These interactions can significantly impact the pharmacokinetics (PK) of co-administered small molecule drugs (SMDs) by modulating drug-metabolising enzyme activity, especially in conditions where proinflammatory cytokines are elevated. In recognition of their clinical importance, recent guidelines from the U.S. Food and Drug Administration (FDA) and the International Council for Harmonisation (ICH) have emphasised a mechanistic, risk-based framework for assessing TP-DIs. These guidelines advocate for a comprehensive understanding of the interaction mechanisms, comedication effects, and patient-specific factors that may influence therapeutic outcomes [1,2].

Among proinflammatory cytokines, interleukin-6 (IL-6) plays a pivotal role in regulating expression and activity of cytochrome P450 (CYP), an enzyme family responsible for the metabolism of a wide range of drugs. Elevated IL-6 levels, as commonly observed in conditions such as autoimmune diseases, cancer, and infections, have been associated with CYP suppression and reduced drug clearance [3,4,5,6]. IL-6 initiates its classic signalling pathway by binding to membrane-bound IL-6 receptors (mIL-6R) expressed on hepatocytes, epithelial cells, and some leukocytes. Upon IL-6 binding to mIL-6R, the signal transducer glycoprotein-130 is recruited, activating downstream pathways involved in the hepatic acute-phase response, thereby downregulating intestinal and hepatic CYP enzyme expression [7]. Blocking IL-6 signalling can reverse this suppression, restore CYP activity, and thereby increase drug metabolism and reduce the exposure of CYP substrate drugs.

Rheumatoid arthritis (RA) is a chronic immune-mediated inflammatory disorder characterised by persistent synovitis and progressive destruction of cartilage and bone. IL-6 is a major mediator of the chronic inflammatory process in RA, contributing to both local joint pathology and systemic manifestations. Serum IL-6 concentrations in RA patients typically range from 10 to 500 pg/mL, with levels occasionally exceeding 1000 pg/mL in severe cases, compared to <5 pg/mL in healthy individuals [8]. Given its central role in disease pathophysiology and its association with systemic inflammation, IL-6 has been a major therapeutic target in RA management. Inhibitors of the IL-6 pathway—targeting the cytokine itself or its receptor—have demonstrated clinical efficacy in suppressing inflammation and reducing disease activity.

Tocilizumab is a humanised monoclonal antibody that binds to both membrane-bound and soluble forms of IL-6R, thereby blocking IL-6-mediated signal transduction [9]. While this blockade results in a paradoxical rise in circulating IL-6 levels, it effectively neutralises IL-6 signalling and ameliorates systemic inflammation [10,11,12]. Tocilizumab is approved for the treatment of moderate to severe active RA in patients with an inadequate response to one or more disease-modifying antirheumatic drugs (DMARDs). It can be administered as monotherapy or in combination with non-biologic DMARDs via either intravenous (IV) or subcutaneous (SC) routes. The clinical study by Schmitt et al. demonstrated that co-administration of simvastatin, a CYP3A4 substrate, with a single IV dose of tocilizumab led to a 57% decrease in simvastatin exposure in RA patients [12]. However, a comprehensive mechanistic framework capable of providing in-depth insights into such interactions across different drugs and clinical settings remains to be established. Previous physiologically based pharmacokinetic (PBPK) models simulating the effect of IL-6 on CYP enzymes have used a semi-mechanistic approach that did not explicitly consider cytokine binding to its receptor, so the effects of an IL-6R antagonist, such as tocilizumab, could not be mechanistically incorporated [8,13,14].

The objectives of this study were to (i) develop a PBPK framework to simulate cytokine receptor antagonist-drug interactions by incorporating competitive receptor binding between endogenous cytokines and therapeutic antagonists, (ii) evaluate the model’s predictive performance by comparing simulated outcomes with clinically observed changes in simvastatin exposure following tocilizumab administration, and (iii) explore the TP-DI liability of tocilizumab with co-administered CYP substrates, including narrow therapeutic index drugs, in RA patients receiving clinically recommended dosing regimens.

## 2. Materials and Methods

### 2.1. Integration of PBPK Models for Simulating TP-DIs

**Figure 1:**
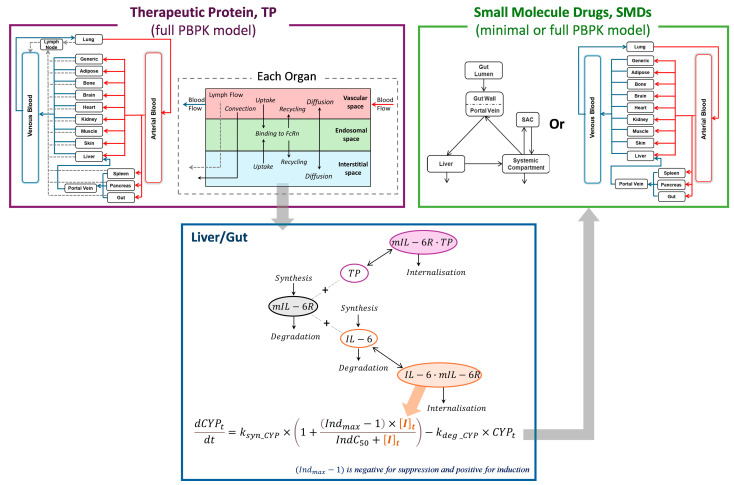
**Schematic of simultaneous modelling of TP and co-administered SMDs to predict IL-6R antagonist-drug interactions.** The disposition of an IL-6R antagonist (e.g., tocilizumab) is described using a full PBPK model for TPs. In the liver and gut interstitial spaces, endogenous IL-6 binds to mIL-6R, forming IL-6/mIL-6R complexes that mediate the suppression of CYP expression and activity under inflammatory conditions. Upon administration of the IL-6R antagonist, it prevents IL-6 from binding to mIL-6R, reduces IL-6/mIL-6R complex formation, and subsequently alleviates CYP suppression. The restoration of CYP enzyme activity increases the metabolism of co-administered SMDs, resulting in decreased drug exposure. The PK of SMDs is captured using either a minimal or full PBPK model.

The structure of the PBPK model used in this study builds upon previously published models for both therapeutic proteins (TPs) and SMDs [15,16,17]. Briefly, the PBPK framework represents the human body as a series of interconnected compartments, each corresponding to a specific organ or tissue, linked by systemic blood flow. These compartments are physiologically structured to reflect organ-specific characteristics relevant to drug distribution and metabolism. For TPs, lymphatic drainage is modelled independently of blood circulation. Each tissue compartment is further divided into vascular, endosomal, and interstitial subspaces to account for mechanisms such as convection, diffusion, and transcytosis involved in protein distribution (Figure 1).

In this study, a mechanistically integrated PBPK framework was developed by combining a full PBPK model for TPs, a minimal or full PBPK model for SMDs, and a cytokine–enzyme interaction model (Figure 1). The framework simulates cytokine dynamics in pathophysiological states, such as RA, where elevated IL-6 levels promote IL-6/mIL-6R complex formation and suppress CYP enzyme expression in liver and gut. The disposition of an IL-6R antagonist (i.e., tocilizumab) was described using the TP PBPK framework, where it competes with endogenous IL-6 for binding to mIL-6R. By occupying mIL-6R, tocilizumab reduces the formation of IL-6/mIL-6R complexes, thereby mitigating IL-6-mediated suppression of CYP enzymes. These dynamic changes in cytokine signalling were quantitatively linked to CYP activity modulation, which subsequently altered the PK of co-administered CYP substrate drugs. The resulting changes in drug exposure were captured using SMD PBPK models, enabling quantitative prediction of TP-DIs through the integrated modelling framework (Figure 1).

#### 2.1.1. Modelling Tocilizumab Profiles Following IV and SC Administration

The input parameters of the tocilizumab PBPK model are summarised in Table 1. Briefly, the distribution of tocilizumab was governed by systemic blood and lymphatic circulation, passive diffusion and convection across endothelial pores, and transcytosis. The latter is driven by endosomal uptake (K_up_), where tocilizumab binds to the neonatal Fc receptor (FcRn), enabling recycling (K_rc_) to the vascular or interstitial space, and protects the antibody from catabolic degradation (CL_cat_). Convection and diffusion of tocilizumab were predicted using the two-pore hypothesis [18,19]. Competition for FcRn binding within the endosomal space was included by simultaneously modelling endogenous IgG and tocilizumab, using parameter values for K_up_, K_rc_, and fraction recycled (FR) based on the estimates for IgG data [20]. The binding of tocilizumab to mIL-6R in the liver and gut was modelled using target-mediated drug disposition (TMDD) with a quasi-equilibrium approximation, parameterised using in vitro binding data [21]. The SC absorption was modelled using a first-order absorption model, where the absorption rate constant (k_a_ = 0.02 h^−1^) and fraction absorbed (f_a_ = 0.3) were fitted to the PK data following a single SC dose of 162 mg [22]. While this f_a_ value adequately captured tocilizumab exposure under SC biweekly (Q2W) dosing, it underpredicted concentrations during SC weekly (QW) dosing. To better capture tocilizumab exposure at steady state following the SC QW regimen, f_a_ was empirically optimised to 0.65, as the underlying mechanisms behind nonlinearity are likely multifactorial and not yet fully understood.

#### 2.1.2. Modelling of Interactions Between Tocilizumab, IL-6, and mIL-6R

The representative ordinary differential equations describing the kinetics of the cytokine receptor antagonist, cytokine, and (membrane-bound) cytokine receptor interactions are presented below to illustrate the model structure, and the full set of equations and assumptions are provided in the Supplementary Materials (Methods Sections A and B).

The free cytokine receptor concentration in tissue interstitial space, [R], is defined in Equation (1):

(1) $$\frac{d[R]}{dt}=k_{syn}^{R}-k_{deg}^{R}\left[R\right]-\left(k_{on}^{RC}\left[C\right]\left[R\right]-k_{off}^{RC}\left[RC\right]\right)-\left(k_{on}^{RLg}\left[R\right]\left[Lg\right]-k_{off}^{RLg}\left[RLg\right]\right)$$

The synthesis of cytokine was assumed to occur exclusively in the plasma, as described by Equation (2):

(2) $$\frac{d{\left[Lg\right]}_{plasma}}{dt}=k_{syn}^{Lg}-k_{deg}^{Lg}{\left[Lg\right]}_{plasma}$$

The free cytokine concentration in tissue interstitial space, [Lg], is defined in Equation (3), where J describes the total flux of cytokine from plasma into interstitial space through diffusion and convection [18,19]:

(3) $$V\frac{d[Lg]}{dt}=J-L\left[Lg\right]-\left(k_{on}^{RLg}\left[R\right]\left[Lg\right]-k_{off}^{RLg}\left[RLg\right]\right)V$$

The cytokine-cytokine receptor complex in tissue interstitial space, [RLg], is defined in Equation (4):

(4) $$\frac{d[RLg]}{dt}={\;k}_{on}^{RLg}\left[R\right]\left[Lg\right]-\left(k_{int}^{RLg}+k_{off}^{RLg}\right)\left[RLg\right]$$

The cytokine receptor antagonist-cytokine receptor complex in tissue interstitial space, [RC], is defined by Equation (5):

(5) $$\frac{d[RC]}{dt}=k_{on}^{RC}[C][R]-(k_{int}^{RC}+k_{off}^{RC})[RC]$$

where C represents the free concentration of the cytokine receptor antagonist in tissue interstitial space, $k_{on}^{RC}$ and $k_{on}^{RLg}$ represent rate constants for the cytokine receptor antagonist and cytokine binding to the cytokine receptor, respectively, $k_{off}$ represents the rate constant for the dissociation receptor complex, $k_{int}$ represents the rate constant for receptor complex internalisation, $k_{deg}$ represents the degradation rate constant, $k_{syn}$ represents the synthesis rate, V represents the tissue interstitial space volume, and L represents the lymph flow rate.

The degradation rate constant of IL-6 (0.269 h^−1^) was estimated based on the half-life of IL-6 obtained from data following a single IV infusion [28]. The interactions between IL-6 and mIL-6R were modelled by a TMDD quasi-equilibrium approximation model (Supplementary Methods Section B). The equilibrium dissociation constant of 8.00 × 10^−5^ µM for IL-6 binding to mIL-6R was obtained from an in vitro study [29]. The rate constant of IL-6/mIL-6R complex internalisation was assumed to be equivalent to the degradation rate constant of mIL-6R (0.277 h^−1^) [30].

#### 2.1.3. Modelling of IL-6/mIL-6R Complex-Driven Suppression of CYP Enzymes

The modulation of CYP enzyme expression was described using an indirect turnover model, where the IL-6/mIL-6R complex dynamically regulates the synthesis rate of CYP enzymes in hepatic and intestinal tissues, as shown in Equation (6):

(6) $$\frac{{dCYP}_{t}}{dt}=k_{syn}\times \left(1+\frac{\left({Ind}_{max}-1\right)\times {\left[I\right]}_{t}}{{IndC}_{50}+{\left[I\right]}_{t}}\right)-k_{deg}\times {CYP}_{t}$$

where ${\left[I\right]}_{t}$ is the IL-6/mIL-6R complex concentration in the gut or liver interstitial space, ${CYP}_{t}$ represents the amount of active CYP enzyme at any given time (t) in the liver or gut, $k_{deg}$ represents the degradation rate constant of the CYP enzyme, $k_{syn}$ represents the synthesis rate of the CYP enzyme, which is equal to $k_{deg}$ multiplied by the basal amount of enzyme in the liver and gut, ${Ind}_{max}$ represents the maximum fold induction/suppression over the vehicle control (>1 = induction; <1 (but >0) = suppression), and ${IndC}_{50}$ represents the effective IL-6 concentration causing half of the maximal suppressive effect.

CYP enzyme suppression parameters, including CYP1A2, CYP2C9, CYP2C19, and CYP3A, were obtained from in vitro studies using primary human hepatocytes treated with IL-6 [31]. The IL-6-related CYP suppression model has been verified against a series of clinical TP-DI studies, as described previously [32].

The baseline suppression of CYP enzymes in RA patients—prior to tocilizumab treatment—was determined by the steady-state concentration of the IL-6/mIL-6R complex in the interstitial spaces of the liver and gut. These values were derived using a steady-state analytical solution of the cytokine–cytokine receptor binding model, as detailed in the Supplementary Materials (Methods Section C).

### 2.2. Virtual Populations

Simulations were performed using a virtual population that represents adult patients with active RA. The demographic, physiological, and biochemical characteristics of this virtual cohort have been detailed previously [32]. The total mIL-6R levels were set at 2.2 nM in the liver and 0.277 nM in the gut, based on expression data in HepG2 cells [33] and the receptor binding assay in Caco-2 cells [34], respectively. The $k_{deg}$ of 0.277 h^−1^ for mIL-6R is based on the reported half-life of 2–3 h [30]. A 30% coefficient of variation (CV) was applied to mean values of mIL-6R levels and $k_{deg}$ to capture typical interindividual variability.

### 2.3. Clinical Data and Simulations

The population-based PBPK simulator (Simcyp Version 23.2, Certara UK Ltd., Sheffield, UK) was used for all simulations. Published PBPK models were used for celecoxib, chloroquine, cyclosporine, ibuprofen, prednisone, simvastatin, and theophylline [35,36,37,38,39]. For simvastatin, the original first-order absorption model was replaced with the advanced dissolution, absorption, and metabolism (ADAM) model to better characterise its oral absorption kinetics [17]. Where available, simulation demographics and dosing regimens were aligned with those reported in corresponding clinical studies. Observed PK data were extracted from the literature using GetData Graph Digitizer v2.26.

#### 2.3.1. Clinical Study Simulations

Study 1: Simulations were performed for 10 trials of 10 RA patients (82.6% female, mean age 52.8 ± 12.5 years) receiving tocilizumab 8 mg/kg intravenously every 4 weeks (Q4W) for 6 doses. Simulated and observed tocilizumab concentration–time profiles were compared [40].

Study 2: Simulations of 10 trials of 15 RA patients (87% female, mean age 54.7 ± 12.4 years) were performed for 162 mg SC tocilizumab administered biweekly (Q2W) over 6 doses. Simulated and observed PK profiles were evaluated [41].

Study 3: Simulations of 10 trials of 14 RA patients (50% female, mean age 58.2 ± 10.8 years) were performed for 162 mg SC tocilizumab administered weekly (QW) for 12 doses, with comparisons to observed concentration–time profiles [41].

Study 4: Simulations of 10 trials of 12 RA patients (assumed 50% female, age range 28 to 72 years) were performed for simvastatin 40 mg orally administered before, 1 week after, and 5 weeks after a single 10 mg/kg IV dose of tocilizumab. Simulated PK data for both tocilizumab and simvastatin were compared against observed clinical data reported by Schmitt et al. [12]. In that study, baseline IL-6 concentrations in RA patients were 51 ± 49 pg/mL (mean ± SD) [12]. To account for clinical heterogeneity and assay variability, simulations were conducted using two representative IL-6 baselines: 50 pg/mL (mean, with a 30% CV) and 100 pg/mL (approximating the upper quartile, with a 30% CV).

#### 2.3.2. Prospective Application Simulations

Simulation of 10 trials of 10 RA patients (50% female, age range 40 to 70 years) were performed over a 12-week treatment period. Three clinically approved dosing regimens of tocilizumab were simulated: 8 mg/kg IV Q4W, 162 mg SC Q2W, or 162 mg SC QW. Each tocilizumab regimen was co-administered with one of the following oral medications: celecoxib at 200 mg twice daily (BID), chloroquine at 300 mg once daily (QD), cyclosporine at 1.25 mg/kg BID, ibuprofen at 200 mg BID, prednisone at 6.5 mg QD, simvastatin at 40 mg QD, or theophylline at 200 mg BID. In these simulations, a baseline IL-6 concentration of 100 pg/mL (mean, with a 30% CV)—approximating the upper quartile reported in RA patient meta-analyses [8,14]—was used. Changes in systemic exposure of the co-administered drugs were assessed as area under the plasma concentration–time curve (AUC) ratio with versus without tocilizumab. For narrow therapeutic index drugs, such as cyclosporine and theophylline, trough concentrations were also evaluated in relation to known therapeutic and toxic thresholds.

#### 2.3.3. Sensitivity Analysis

To evaluate the impact of cytokine variability and cytokine receptor genetic polymorphisms on TP-DI predictions, sensitivity analyses were performed on baseline IL-6 and mIL-6R levels within clinically relevant ranges. For IL-6, simulations explored a broader physiological range of 10–1000 pg/mL, based on reported levels in RA patients [8]. Changes in simvastatin exposure (AUC and C_max_ ratios) following a single tocilizumab dose were simulated in virtual RA subjects, aligned with the design of the clinical study [12]. The IL-6R (Asp358Ala) polymorphism is known to enhance shedding of mIL-6R. Reductions of 28% and 56% in mIL-6R levels were simulated to represent heterozygous and homozygous carriers, respectively [42].

## 3. Results

### 3.1. Prediction of Tocilizumab Concentration–Time Profiles Across IV and SC Dose Regimens

**Figure 2:**
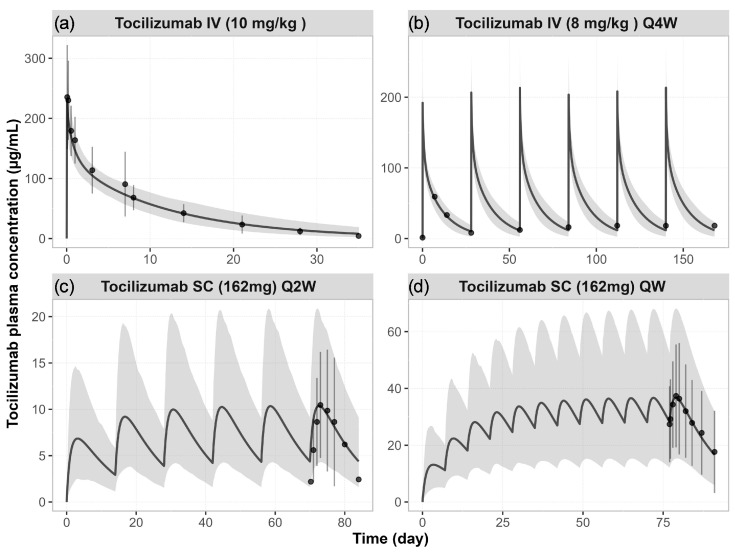
**Simulated vs. observed plasma concentration–time profiles of tocilizumab in RA patients.** Simulated (lines) and observed (data points [12,40,41]) mean plasma concentration–time profiles of tocilizumab following (**a**) a single IV dose of 10 mg/kg, (**b**) IV doses of 8 mg/kg Q4W, (**c**) SC doses of 162 mg Q2W, and (**d**) SC doses of 162 mg QW. The shaded areas represent the 5th to 95th percentiles of total virtual populations. The error bars represent the standard deviation of the observed data.

The simulated concentration–time profiles of tocilizumab in RA patients were in good agreement with observed clinical data following both single and multiple doses, including IV administration at 10 mg/kg (single dose) and 8 mg/kg (Q4W), as well as SC administration at 162 mg Q2W and 162 mg QW (Figure 2). Across all regimens, the model was able to capture the time course and magnitude of tocilizumab exposure. The simulated population variability covered most of the observed interindividual variability (Figure 2).

### 3.2. Prediction of the Time Course of TP-DIs Between Tocilizumab and Simvastatin

**Figure 3:**
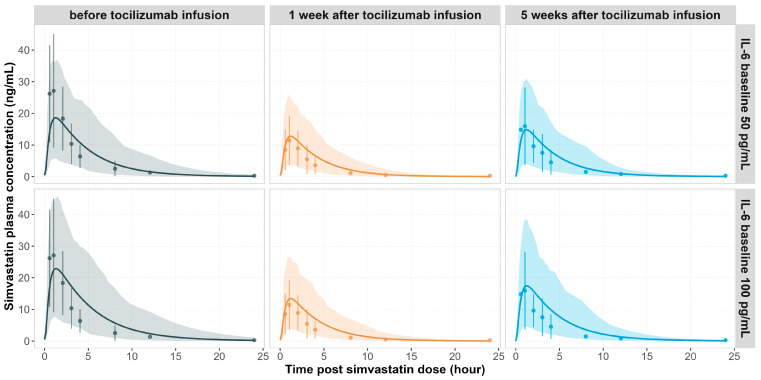
**Simulated vs. observed plasma concentration-time profiles of simvastatin in RA patients after a single IV administration of tocilizumab (10 mg/kg).** Panels show simulated (lines) and observed (data points [12]) mean plasma concentration–time profiles of simvastatin before (left panel), 1 week after (middle panel), and 5 weeks after (right panel) a single IV administration of 10 mg/kg tocilizumab. Simulations were performed using baseline IL-6 concentrations of 50 pg/mL (top row) and 100 pg/mL (bottom row), respectively. The shaded areas represent the 5th to 95th percentiles of total virtual populations. The error bars represent the standard deviation of the observed data.

The PBPK model captured the time course of simvastatin exposure changes observed clinically before and after a single IV infusion of tocilizumab 10 mg/kg, using the baseline IL-6 levels of 50 and 100 pg/mL, respectively (Figure 3). The corresponding concentration–time profile of tocilizumab is shown in Figure 2a.

Prior to tocilizumab treatment, elevated IL-6 levels suppressed CYP3A4 activity in RA patients, leading to reduced simvastatin clearance and increased plasma exposure of simvastatin (Figure 3, left panel). Following tocilizumab administration, IL-6 signalling was diminished due to IL-6 receptor blockade by tocilizumab. The CYP3A4 activity was restored. This resulted in reduced simvastatin exposure one week post-treatment (Figure 3, middle panel). The observed geometric mean ratios (GMRs) for simvastatin C_max_ and AUC_inf_ were both 0.43. At a baseline IL-6 of 50 pg/mL, the predicted GMRs were 0.66 and 0.60 for C_max_ and AUC_inf_, respectively (predicted over observed, P/O: 1.53 and 1.39). Using 100 pg/mL as the baseline IL-6, the model predicted GMRs of 0.56 and 0.47 (P/O: 1.30 and 1.09), aligning more closely with observed values.

Five weeks after tocilizumab infusion, as drug concentrations declined, a gradual return of IL-6 signalling was evident, leading to partial re-suppression of CYP3A4 and a corresponding increase in simvastatin exposure (Figure 3, right panel). Observed GMRs for C_max_ and AUC_inf_ were 0.60 and 0.61, respectively. The model predicted GMRs of 0.77 and 0.71 (P/O: 1.28 and 1.16) for the 50 pg/mL IL-6 baseline scenario, and 0.72 and 0.65 (P/O: 1.20 and 1.07) for the 100 pg/mL IL-6 baseline, showing improved prediction at higher IL-6 baseline levels.

Across all simulated scenarios, all predicted values fell within a 2-fold error of the observed data, with 7 out of 8 within 1.5-fold and 4 out of 8 within the 0.8–1.25 range, indicating good predictive performance.

The sensitivity analysis across a wide IL-6 baseline range (10–1000 pg/mL) observed in RA patients demonstrated a nonlinear relationship between the IL-6 baseline level and simvastatin exposure changes one week after a single IV administration of 10 mg/kg tocilizumab (Figure S1). As baseline IL-6 decreased from 100 to 10 pg/mL, the predicted AUC ratios increased from 0.47 to 0.85, while it reached a plateau of ~0.35 at 500 pg/mL and above. A similar trend was observed for C_max_ ratios, which increased from 0.56 at 100 pg/mL to 0.88 at 10 pg/mL, and plateaued around 0.43 at higher IL-6 levels. Sensitivity analyses were also performed on mIL-6R levels to account for receptor polymorphisms. Simulated reductions in mIL-6R levels of 28% and 56%, representing heterozygous and homozygous IL-6R Asp358Ala variants, resulted in minimal changes (<1.5%) in simvastatin AUC and C_max_ ratios.

### 3.3. Mechanistic Insights into IL-6 Signalling and CYP3A4 Modulation Following Tocilizumab Administration

**Figure 4:**
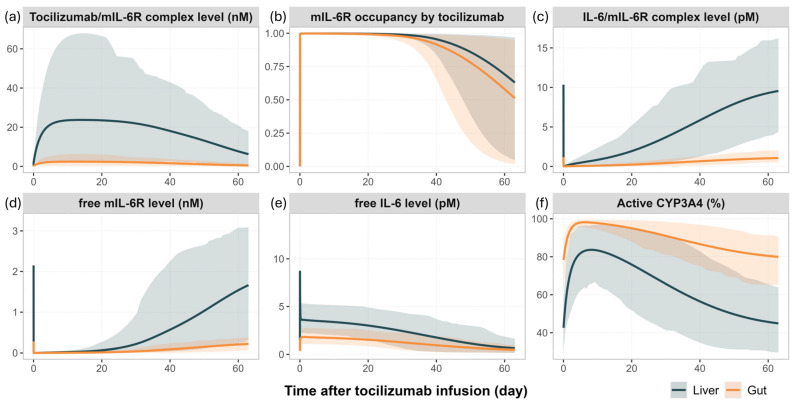
**Simulated time course of cytokine signalling and CYP3A4 regulation in the liver and gut following a single IV administration of tocilizumab (10 mg/kg) in RA patients.** Simulated (lines) mean profiles include (**a**) tocilizumab/mIL-6R complex levels, (**b**) mIL-6R occupancy by tocilizumab, (**c**) IL-6/mIL-6R complex levels, (**d**) free mIL-6R levels, (**e**) free IL-6 levels, and (**f**) active CYP3A4 enzyme levels (fold change from baseline) in the liver and gut. Simulations were performed in virtual RA patients with a baseline IL-6 level of 100 pg/mL. Simulated results are shown for the liver (grey) and gut (orange). The shaded areas represent the 5th to 95th percentiles of total virtual populations.

Figure 4 illustrates the model predicted temporal changes in tocilizumab—mIL-6R interactions, IL-6 signalling dynamics, and CYP3A4 activity in the liver and gut following a single IV administration of tocilizumab (10 mg/kg).

Shortly after dosing, tocilizumab bound rapidly to mIL-6R in both the liver and gut, reaching maximal target occupancy (~100%, Figure 4b) and forming high levels of tocilizumab/mIL-6R complexes (Figure 4a). This receptor blockade significantly reduced the availability of free mIL-6R (Figure 4d), suppressing the formation of IL-6/mIL-6R complex (Figure 4c), the primary driver of CYP3A4 downregulation. As a result, the predicted CYP3A4 activity increased rapidly in both the liver and gut, particularly within the first week post-dose (Figure 4f). In parallel, the blockade of receptor-mediated clearance of IL-6 by tocilizumab led to an accumulation of free IL-6 in both tissues (Figure 4e). Over time, as tocilizumab concentrations declined, mIL-6R occupancy by tocilizumab decreased, leading to a gradual increase of IL-6/mIL-6R complexes and a progressive decline in CYP3A4 activity.

These temporal patterns are consistent with the dynamic changes in simvastatin exposure reported in clinical studies and captured by the model, as described in Section 3.2. The magnitude of simulated IL-6/mIL-6R complex formation and CYP3A4 suppression was consistently greater in the liver compared to the gut, reflecting tissue-specific differences in receptor expression and cytokine-driven enzyme regulation.

### 3.4. Prospective Predictions of TP-DIs in RA Patients with Comedications of Tocilizumab and CYP Substrate Drugs

To demonstrate the translational utility of the PBPK framework, prospective simulations were performed in virtual RA patients receiving clinically relevant regimens of tocilizumab in combination with commonly prescribed CYP substrate drugs. Given that trial-based clinical TP-DI data are currently limited to simvastatin following a single IV dose of tocilizumab, these prospective simulations could support model-informed risk assessment across more real-world scenarios. The three simulated tocilizumab regimens included 8 mg/kg IV Q4W, 162 mg SC Q2W, and 162 mg SC QW. Each tocilizumab regimen was co-administered with one of the following oral medications: celecoxib (200 mg BID), chloroquine (300 mg QD), cyclosporine (1.25 mg/kg BID), ibuprofen (200 mg BID), prednisone (6.5 mg QD), simvastatin (40 mg QD), or theophylline (200 mg BID).

**Figure 5:**
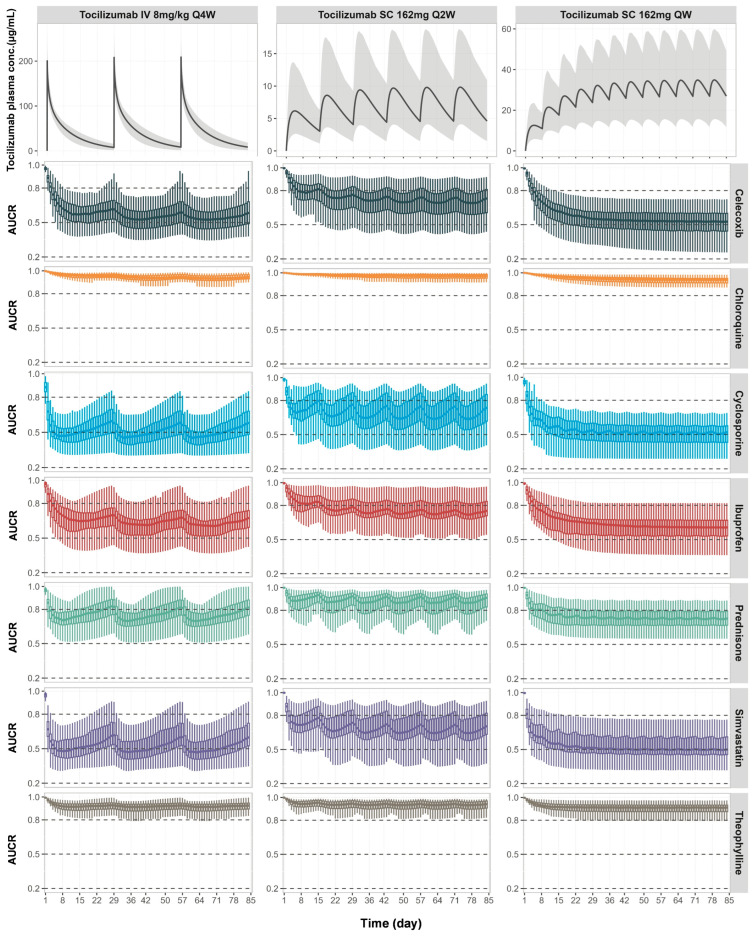
**Predicted changes in exposure of co-administered CYP substrate drugs during tocilizumab treatment across multiple dosing regimens.** Simulated mean plasma concentration-time profiles of tocilizumab (top row) and corresponding AUC ratios (AUCR, post-/pre-tocilizumab) for co-administered CYP substrate drugs (box plots in rows below) in RA patients receiving tocilizumab 8 mg/kg IV Q4W (left), 162 mg SC Q2W (middle), or 162 mg SC QW (right). In the top row, solid lines represent the population mean and grey shaded areas represent the 5th to 95th percentiles of total virtual populations. Box plots represent simulated AUCR values over a 12-week dosing period for celecoxib, chloroquine, cyclosporine, ibuprofen, prednisone, simvastatin, and theophylline. The central bold line represents the median, box edges represent the 25th and 75th percentiles, and whiskers represent the 5th and 95th percentiles of the total virtual population.

Simulations predicted varying degrees of interactions depending on the CYP substrate and the tocilizumab regimen (Figure 5). Moderate reductions in drug exposure occurred for simvastatin, cyclosporine, and celecoxib, reflecting changes in their CYP3A4- and CYP2C9-mediated clearance and the dynamic restoration of enzyme activity following IL-6 suppression. Mild-to-moderate interactions were simulated for ibuprofen and prednisone. These changes were more pronounced with the QW and Q2W SC regimens, which maintained higher tocilizumab concentrations and more sustained IL-6 receptor blockade compared to the IV Q4W regimen. Minimal changes in drug exposure were predicted for chloroquine and theophylline. The model also predicted the temporal variability in exposure due to periodic dosing, especially for drugs with short half-lives (e.g., ibuprofen and simvastatin), where drug levels fluctuate alongside tocilizumab-mediated modulation of enzyme activity.

**Figure 6:**
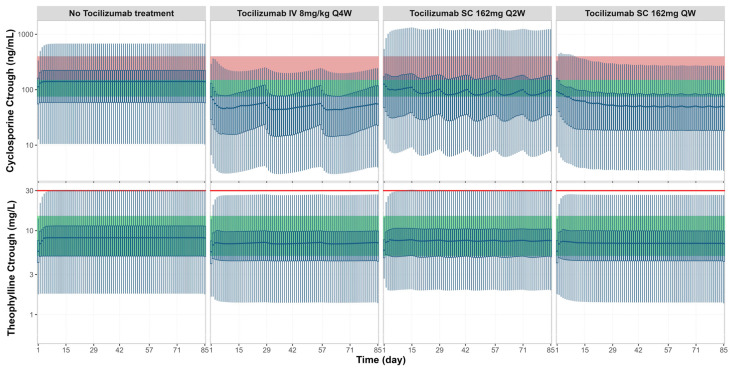
**Simulated cyclosporine and theophylline trough concentrations without and with tocilizumab treatment across three clinical regimens.** Box plots represent the dynamic changes of predicted trough concentrations for cyclosporine (top row, ng/mL) and theophylline (bottom row, mg/L) in RA patients under four conditions: no tocilizumab treatment and co-administration with tocilizumab treatment of 8 mg/kg IV Q4W, 162 mg SC Q2W, or 162 mg SC QW. The central bold line represents the median, box edges represent the 25th and 75th percentiles, and whiskers represent the 5th and 95th percentiles of the total virtual population. Green shaded areas represent the known therapeutic windows, and red shaded areas and red lines indicate toxicity thresholds [43,44].

Additional simulations were performed to assess the potential interaction risks between tocilizumab and narrow therapeutic index drugs, namely, cyclosporine and theophylline, by evaluating predicted trough concentrations relative to therapeutic windows and toxicity thresholds (Figure 6). For cyclosporine, tocilizumab co-administration resulted in a consistent reduction in trough concentrations across all three dosing regimens. In majority of virtual RA patients, the predicted post-treatment trough levels fell below the therapeutic window (green zone), particularly under the IV Q4W and SC QW regimens. For theophylline, trough concentrations showed minimal change across all tocilizumab dosing regimens. The trough concentrations remained within the therapeutic window (green zone) and below the toxicity threshold (red line), indicating a low risk of clinically meaningful interaction under the simulated conditions.

## 4. Discussion

In this study, a mechanistic PBPK model was developed to simulate TP-DIs involving cytokine antagonists, with tocilizumab used as a representative IL-6 receptor antagonist. The model incorporated cytokine–receptor dynamics, TMDD, and cytokine-mediated modulation of CYP enzyme activity, enabling prediction of drug interactions under inflammatory conditions and during therapy with cytokine antagonists. The simulated concentration–time profiles of tocilizumab following both IV and SC administration were consistent with clinical observations. Moreover, the PBPK model quantitatively captured the clinically observed time-dependent changes in simvastatin exposure following tocilizumab treatment [12]. The model was further applied to prospectively assess the TP-DI liability of comedications that are CYP substrates, including drugs with narrow therapeutic indices, in real-world dosing scenarios.

An important pharmacological phenomenon captured by the model is the elevation of IL-6 levels following tocilizumab treatment (Figure 4). This effect has been consistently reported in clinical studies and is attributed to inhibition of IL-6 receptor-mediated clearance, rather than increased cytokine production [10,12,45]. By binding to IL-6 receptors, tocilizumab prevents IL-6 internalisation and degradation, leading to accumulation of free IL-6 in circulation. Schmitt et al. reported that serum IL-6 levels increased by 5.13-fold (±4.74) on day 2, remained elevated at 4.61-fold (±5.01) through day 29, and gradually returned toward baseline around day 57 following a single IV dose of tocilizumab [12]. A similar temporal pattern was reproduced in the PBPK model: simulated free IL-6 concentrations in the gut and liver interstitial spaces increased approximately 5- to 10-fold and followed a comparable time course, peaking early, and declined as tocilizumab concentrations decreased (Figure 4e). Importantly, this rise in IL-6 levels does not indicate a worsening of inflammation or higher IL-6 production. Clinical studies have shown that IL-6 mRNA expression in peripheral blood mononuclear cells remained unchanged following tocilizumab treatment, while serum C-reactive protein levels and other downstream inflammatory markers normalised, indicating effective suppression of IL-6 signalling [10]. This dissociation between cytokine concentration and the enzyme suppression effect is consistent with model predictions, showing restored CYP3A4 activity and reduced simvastatin exposure after tocilizumab treatment (Figure 3 and Figure 4f). Together, these results support the concept that cytokine receptor occupancy, rather than circulating cytokine levels, is the primary determinant of pharmacodynamic outcomes, and reinforce the interpretation of elevated IL-6 as a marker of effective receptor blockade, not disease progression in this scenario.

In RA, IL-6 is predominantly produced at sites of inflammation, such as the synovial membrane and activated immune cells, with systemic IL-6 concentrations reflecting this increased peripheral production [46]. The current model assumes IL-6 synthesis occurs exclusively in plasma, without explicitly representing tissue-specific production. While local IL-6 synthesis in the liver may contribute to total exposure, there is no evidence suggesting that hepatic production is the primary driver of elevated IL-6 levels in RA. Moreover, quantitative human data on IL-6 concentrations within liver tissue during inflammation are lacking. IL-6 is also known to exhibit circadian rhythm. In a study of 13 RA patients, serum IL-6 concentrations peaked in the morning (~90 pg/mL), declined to ~40 pg/mL in the afternoon, and stabilised around 30 pg/mL from evening to night [47]. While these fluctuations were not explicitly modelled, the suppressive effect of IL-6 on CYP expression is governed not only by cytokine concentration, but also receptor binding kinetics and turnover rates of CYP enzymes. Given that the half-lives of CYPs (CYP1A2, CYP2C9, CYP2C19, and CYP3A) evaluated in this study exceeded 24 h, transient IL-6 fluctuations are unlikely to significantly alter enzyme levels over a single circadian cycle. Moreover, once tocilizumab is administered, it rapidly and effectively saturates IL-6 receptors, sustainably neutralising IL-6 signalling (Figure 4). Thus, circadian variation of IL-6 is not expected to have a significant impact on TP-DI outcomes. Nonetheless, incorporation of liver-specific IL-6 synthesis and circadian IL-6 patterns could be considered in future model refinements, accounting for additional biological complexity.

The prospective simulation results underscore both the clinical relevance of timing and the substrate-dependent nature in cytokine-mediated TP-DIs. The magnitude and temporal pattern of CYP modulation varied across different tocilizumab dosing regimens (Figure 5). The IV Q4W and SC Q2W regimens produced more fluctuations in CYP activity and AUC ratio changes over time, corresponding to the periodic rise and fall of tocilizumab concentrations between doses. In contrast, the SC QW regimen maintained more stable tocilizumab levels, resulting in sustained IL-6R blockade and a consistent degree of CYP reactivation. These differences underscore the need to consider not just the drug exposure magnitude but also the frequency and stability of receptor occupancy when evaluating TP-DI risk. This has meaningful implications for drugs with narrow therapeutic indices, such as cyclosporine, which is used in RA management [48]. Clinical guidelines recommend an initial cyclosporine dose of 1.25 mg/kg BID in RA patients, and our simulations indicated that this dose generally achieved therapeutic trough concentrations prior to tocilizumab initiation; however, a subset of patients exhibited trough levels above the toxic threshold, likely reflecting baseline variability in IL-6-mediated CYP3A4 suppression (Figure 6). Following tocilizumab administration, the restoration of CYP3A4 activity increased cyclosporine clearance, leading to a notable decline in trough concentrations. As a result, cyclosporine exposure fell below the therapeutic range in many virtual patients, potentially compromising efficacy. These observations emphasise the need for therapeutic drug monitoring and individualised dosing strategies when initiating or discontinuing cytokine antagonists, particularly for drugs that rely on narrow exposure margins for safety and efficacy. The simulations also revealed substrate-dependent differences in TP-DI magnitude, consistent with previous reports and in vitro findings [31,32]. Substrates primarily metabolised by CYP3A4 and CYP2C9, such as simvastatin, cyclosporine, ibuprofen, and celecoxib, exhibited the most pronounced exposure changes, while CYP1A2 (e.g., for theophylline) showed minimal interaction. This differential response may reflect the underlying cytokine-specific mechanisms that regulate drug-metabolising enzymes. IL-6 suppresses CYP expression through multiple pathways, including activation of JAK/STAT3 signalling, which interferes with the transcriptional activity of key hepatic nuclear receptors, such as PXR and CAR, essential for CYP3A4 and CYP2C9 gene regulation [49,50,51].

In addition to CYP enzymes, cytokine-mediated regulation extends to other drug-metabolising enzymes and transporters, although this area remains less quantitatively defined. In vitro studies—including both hepatocyte monocultures and the advanced hepatocyte–Kupffer cell co-culture system—have shown that proinflammatory cytokines such as IL-6 and IL-1β downregulate several phase II enzymes (e.g., UGT1A1, UGT2B7, and SULTs) as well as hepatic transporters (e.g., OATP1B1, MRP2, and BSEP) [52,53]. Compared to the consistent downregulation by IL-6, IL-1β exhibits more variable effects, with responses varying across donors and experimental systems [52,53]. In addition, there are no clinically documented TP-DIs involving IL-1β modulators, such as anakinra, canakinumab, rilonacept, or gevokizumab, to date. Therefore, while IL-1β may play a role in CYP regulation in vitro, its clinical relevance remains uncertain.

Nevertheless, these in vitro findings support the hypothesis that cytokine-mediated drug interactions may extend beyond CYP enzymes and IL-6 pathways. While our model focused on IL-6-mediated CYP suppression, it is inherently extensible to other cytokines (e.g., IL-1β and TNF-α) and non-CYP clearance mechanisms once quantitative and translational data become available to parameterise and verify the performance of the model. As biologics targeting various cytokines continue to expand across rheumatology, dermatology, gastroenterology, and oncology, a quantitative, receptor-occupancy-based PBPK approach offers a valuable platform for assessing TP-DI liability beyond IL-6. Such adaptability is particularly important for patients receiving comedications or narrow therapeutic index drugs, enabling precision dosing strategies in complex inflammatory disease settings.

## 5. Conclusions

This study demonstrated the utility of PBPK modelling in providing a mechanistic understanding of cytokine antagonist-drug interactions, using tocilizumab as a representative IL-6R inhibitor. By linking cytokine–receptor dynamics to CYP enzyme modulation, the model captured clinically observed TP-DIs and highlighted the importance of timing, dose regimen, and patient-specific factors. This framework supports informed therapeutic decision-making and enables precision dosing strategies to optimise patient care in inflammatory conditions.

## Figures and Tables

**Table 1:** Tocilizumab PBPK model input.

Parameters	Value	Reference/Note
Molecular Weight (g/mol)	148,000	Drug label [23]
**Subcutaneous Absorption**		
First-Order Absorption Model		
k_a_ (1/h)	0.02	Fitted to PK data after a single SC dose of 162 mg [22]
f_a_	0.3	
**Distribution**		
Distribution Model	Full PBPK	
K_up_ (1/h)	0.0298	[24]
K_rc_ (1/h)	16.7	Fitted based on endogenous IgG data [20]
FR	1	Assumed FR = 1 for all organs except adipose, muscle, and spleen, where FR = 0
K_D,Tocilizumab-FcRn_ (µM)	0.46	[25]
**Elimination**		
CL_cat_ (L/h)	0.0175	Fitted based on endogenous IgG data [20]
CL_add_ (L/h)	0.009	Optimised to recover the PK data after an IV dose of 4 mg/kg [26]
**TMDD Parameters**		
TMDD Model	Quasi-equilibrium approximation	
Drug Target name	mIL-6R	
K_D_Tocilizumab-mIL-6R_ (µM)	1.96 × 10^−4^	[27]
k_int_Tocilizumab/mIL-6R complex_ (1/h)	0.0186	[27]

k_a_: First-order absorption rate constant; f_a_: fraction absorbed; K_up_: uptake rate into endosomal space via fluid phase endocytosis; K_rc_: recycling rate of FcRn-drug complex from endothelial space; K_D_: equilibrium dissociation constant; FR: fraction recycled of FcRn-bound drug; k_int_: rate constant for drug-target complex internalisation; CL_cat_: catabolic clearance; CL_add_: unspecified additional systemic clearance.

## Data Availability

This study did not generate new data. All simulation results are described within the article and its Supplementary Materials.

## Supplementary Materials

The supporting information can be downloaded at: https://www.mdpi.com/article/10.3390/pharmaceutics17070896/s1. Figure S1: Population sensitivity analysis of baseline IL-6 concentrations (10–1000 pg/mL) on the AUC and C_max_ ratios of simvastatin following 1-week tocilizumab treatment.
