# Supplementary material for: Mechanistic Insights into Cytokine Antagonist-Drug Interactions: A Physiologically Based Pharmacokinetic Modelling Approach with Tocilizumab as a Case Study
Source: Pharmaceutics. 2025 Jul 10;17(7):896. doi: 10.3390/pharmaceutics17070896 (PMC12297905; doi:10.3390/pharmaceutics17070896)
Supplement: Supplementary file 1 [file pharmaceutics-17-00896-s001.zip › pharmaceutics-3705369-supplementary.pdf]

Mechanistic Insights into Cytokine Antagonist-Drug Interactions: a Physiologically-Based Pharmacokinetic Modelling Approach with Tocilizumab as a Case Study

Xian Pan, Cong Liu, Felix Stader, Abdallah Derbalah, Masoud Jamei, and Iain Gardner
Certara Predictive Technologies Division, Certara UK Limited, Sheffield, United Kingdom

Supplementary materials

Methods

This supplementary methods section provides the mathematical framework for modelling cytokine receptor antagonist therapeutic protein (TP), cytokine, and (membrane-bound) cytokine receptor kinetics. Sections A and B present ordinary differential equations for full dynamic models and dynamic models with quasi-equilibrium (QE) approximations used in the physiologically-based pharmacokinetic (PBPK) simulations. Section C describes the steady-state solution used for determining initial conditions.

A. Full Dynamic Model of TP–Cytokine–Cytokine Receptor Interactions

When the interactions between TP, cytokine, and (membrane-bound) cytokine receptor is described by full dynamic models, the following expressions and equations are applied.

1) Free cytokine receptor concentration in tissue interstitial space is defined in equation (1)

$$\frac{d[R]_{I,k}}{dt} = k^{R}_{syn,k} - k^{R}_{deg,k}[R]_{I,k} - (k^{RC}_{on,k}C_{I,k}[R]_{I,k} - k^{RC}_{off,k}[RC]_{I,k}) - (k^{RLg}_{on,k}[R]_{I,k}[Lg]_{I,k} - k^{RLg}_{off,k}[RLg]_{I,k}) \quad (1)$$

2) Total cytokine receptor concentration in tissue interstitial space is defined in equation (2)

$$[R]_{I,k,tot} = [R]_{I,k} + [RLg]_{I,k} + [RC]_{I,k} \quad (2)$$

3) Free cytokine concentration in interstitial space for tissue is defined in equation (3)

$$V_{I,k}\frac{d[Lg]_{I,k}}{dt} = J_{V,k}([Lg]_{V,k}, [Lg]_{I,k}) - L_k[Lg]_{I,k} - (k^{RLg}_{on,k}[R]_{I,k}[Lg]_{I,k} - k^{RLg}_{off,k}[RLg]_{I,k})V_{I,k} \quad (3)$$

4) Free cytokine concentration in the tissue vascular space is defined by equation (4)

$$\frac{d[Lg]_{V,k}}{dt} = k^{Lg}_{syn} - k^{Lg}_{deg}[Lg]_{V,k} \quad (4)$$

Where $J_{V,k}([Lg]_{V,k}, [Lg]_{I,k})$ describes the total flux of cytokine from vascular into interstitial space through both diffusion and convection via small and large pores [1,2]

5) Total cytokine concentration in tissue interstitial space is defined in equation (5)

$$[Lg]_{I,\,k,total} = [Lg]_{I,k} + [RLg]_{I,k} \quad (5)$$

6) Cytokine-cytokine receptor complex in tissue interstitial space is defined in equation (6)

$$\frac{d[RLg]_{I,k}}{dt} = k_{on,k}^{RLg}[R]_{I,k}[Lg]_{I,k} - (k_{int,k}^{RLg} + k_{off,k}^{RLg})[RLg]_{I,k} \qquad (6)$$

7) TP-cytokine receptor complex in tissue interstitial space is defined by equation (7)

$$\frac{d[RC]_{I,k}}{dt} = k_{on,k}^{RC}C_{I,k}[R]_{I,k} - (k_{int,k}^{RC} + k_{off,k}^{RC})[RC]_{I,k} \qquad (7)$$

Variable Definitions:

- k = liver or gut
- $C_{I,k}$ = free concentration of TP (e.g., tocilizumab) in interstitial space
- $R_{I,k}$ = free cytokine receptor (e.g., mIL-6R) concentration in interstitial space
- $Lg_{I,k}$ = free cytokine (e.g, IL-6) concentration in interstitial space
- $Lg_{V,k}$ = free cytokine concentration in tissue vascular space
- $RC_{I,k}$ = concentration of TP-cytokine receptor complex in interstitial space
- $RLg_{I,k}$ = concentration of cytokine-cytokine receptor complex in interstitial space
- k_{on}^{RC} = Rate constant for TP binding to cytokine receptor
- k_{on}^{RLg} = Rate constant for cytokine binding to cytokine receptor
- k_{off}^{RC} = Rate constant for dissociation of TP-cytokine receptor complex
- k_{off}^{RLg} = Rate constant for dissociation of cytokine-cytokine receptor complex
- k_{int}^{RLg}= Rate constant for cytokine-cytokine receptor complex internalisation
- k_{int}^{RC}= Rate constant for TP-cytokine receptor complex internalisation
- k_{deg}^{R} = degradation rate constant for cytokine receptor
- k_{syn}^{R} = cytokine receptor synthesis rate
- k_{deg}^{Lg} = degradation rate constant for cytokine
- k_{syn}^{Lg} = cytokine synthesis rate
- $R_{max,Lg,SS}$ = steady-state cytokine concentration in plasma
- $k_{syn}^{Lg} = k_{deg}^{Lg} \times R_{max,Lg,SS}$
- $V_{I,k}$ = interstitial space volume
- L_k = lymph flow rate

Initial Conditions

- $[R]_{I,k,tot}\ (t = 0) =$ total (membrane-bound) cytokine receptor level.
- $[Lg]_{V,k}(t = 0) = R_{max,Lg,SS}$, steady-state cytokine concentration in plasma
- $[RC]_{I,k}\ (t = 0) = 0$
- $[RLg]_{I,k}\ (t = 0)$ is calculated using steady-state solution (see section C)
- $[R]_{I,k}\ (t = 0)$ is calculated using steady-state solution (see section C)
- k_{syn}^{R} is calculated using steady-state solution (see section C)

B. Dynamic model with Quasi-Equilibrium (QE) approximation for TP–Cytokine–Cytokine Receptor Interactions

When the interactions between TP, cytokine, and (membrane-bound) cytokine receptor are assumed to be at quasi-equilibrium, the following expressions and equations are applied.

1) Total cytokine receptor concentration in tissue interstitial space is described by equation (8)

$$\frac{d[R]_{I,k,tot}}{dt} = k_{syn}^{R} - k_{deg}^{R}[R]_{I,k,tot} + [R]_{I,k}\left[\left(k_{deg}^{R} - k_{int}^{RC}\right)\frac{C_{I,k,tot}}{[R_k] + K_D^{RC}} + \left(k_{deg}^{R} - k_{int}^{RLg}\right)\frac{[Lg]_{I,k,tot}}{[R_k] + K_D^{RLg}}\right] \quad (8)$$

2) Total cytokine concentration in tissue interstitial space is described by equation (9)

$$V_{I,k}\frac{d[Lg]_{I,k,tot}}{dt} = J_{V,k}\left([Lg]_{V,k}, [Lg]_{I,k}\right) - L_k[Lg]_{I,k} - k_{int,k}^{RLg}[RLg]_{I,k}V_{I,k} \quad (9)$$

3) TP-cytokine receptor complex concentration in tissue interstitial space is described by equation (10)

$$[RC]_{I,k} = \frac{[R]_{I,k} \times C_{I,k,tot}}{[R]_{I,k} + K_D^{RC}} \quad (10)$$

4) Cytokine-cytokine receptor complex concentration in tissue interstitial space is described by equation (11)

$$[RLg]_{I,k} = \frac{[R]_{I,k} \times [Lg]_{I,k,tot}}{[R]_{I,k} + K_D^{RLg}} \quad (11)$$

5) Deriving free concentrations of TP, cytokine and cytokine receptor in tissue interstitial space

Total concentrations of TP, cytokine receptor and cytokine in tissue interstitial space are defined by equations (12) to (14):

$$C_{I,k,tot} = C_{I,k} + [RC]_{I,k} \quad (12)$$

$$[R]_{I,k,tot} = [R]_{I,k} + [RLg]_{I,k} + [RC]_{I,k} \quad (13)$$

$$[Lg]_{I,k,tot} = [Lg]_{I,k} + [RLg]_{I,k} \quad (14)$$

Substitute $[RC]_{I,k}$ and $[RLg]_{I,k}$ in equations (12) to (14) from QE expression (equations (10) and (11)):

$$C_{I,k,tot} = C_{I,k} + \frac{[R]_{I,k} \times C_{I,k,tot}}{[R]_{I,k} + K_D^{RC}} \quad (15)$$

$$[R]_{I,k,tot} = [R]_{I,k} \times (1 + \frac{[Lg]_{I,k,tot}}{[R]_{I,k} + K_D^{RLg}} + \frac{C_{I,k,tot}}{[R]_{I,k} + K_D^{RC}}) \quad (16)$$

$$[Lg]_{I,k,tot} = [Lg]_{I,k} + \frac{[R]_{I,k} \times [Lg]_{I,k,tot}}{[R]_{I,k} + K_D^{RLg}} \quad (17)$$

To solve for free fractions of cytokine receptor, cytokine, and TP:

Define

$\alpha_{C,k} = \frac{C_{I,k,tot}}{[R]_{I,k,tot}}$, $\alpha_{Lg,k} = \frac{[Lg]_{I,k,tot}}{[R]_{I,k,tot}}$, $\beta_{C,k} = \frac{K_D^{RC}}{[R]_{I,k,tot}}$, $\beta_{Lg,k} = \frac{K_D^{RLg}}{[R]_{I,k,tot}}$, $f_{C,k} = \frac{C_{I,k}}{C_{I,k,tot}}$, $f_{R,k} = \frac{[R]_{I,k}}{[R]_{I,k,tot}}$, $f_{Lg,k} = \frac{[Lg]_{I,k}}{[Lg]_{I,k,tot}}$,

The equation (16) can be transformed to equation (18) and Newton iteration is used to solve for the free fractions of cytokine receptor $f_{R,k}$

$$1 = f_{R,k} \times (1 + \frac{\alpha_{Lg,k}}{f_{R,k}+\beta_{Lg,k}} + \frac{\alpha_{C,k}}{f_{R,k}+ \beta_{C,k}}) \tag{18}$$

Then free fraction of TP and cytokine ($f_{C,k}$ and $f_{Lg,k}$) can be calculated using equations (19)and (20):

$$f_{C,k} = 1 - \frac{[R]_{I,k}}{[R]_{I,k} + K_D^{RC}} \tag{19}$$

$$f_{Lg,k} = 1 - \frac{[R]_{I,k}}{[R]_{I,k} + K_D^{RLg}} \tag{20}$$

Thus, free concentrations of TP, cytokine receptor and cytokine concentrations are derived using equations (21) to (23)

$$C_{I,k} = f_{C,k} \cdot [C]_{I,k,tot} \tag{21}$$

$$[R]_{I,k} = f_{R,k} \cdot [R]_{I,k,tot} \tag{22}$$

$$[Lg]_{I,k} = f_{Lg,k} \cdot [Lg]_{I,k,tot} \tag{23}$$

Variable definitions:

- K_D^{RC} = equilibrium dissociation constant for the cytokine receptor and TP
- K_D^{RLg} = equilibrium dissociation rate constant for the cytokine receptor and the cytokine
- $C_{I,k,tot}$ = total TP concentration in tissue interstitial space
- The rest variables have been defined in section A

Initial conditions:

- $C_{I,k,tot}(t = 0) = 0$
- $[R]_{I,k,tot}(t = 0)$ is calculated using steady-state solution (see section C)
- $[Lg]_{I,k,tot}(t = 0)$ is calculated using steady-state solution (see section C)

C. Steady-state solution for initial conditions using QE approximation

1) Steady-state balance for total cytokine in tissue interstitial space
   At steady-state, equation (9) becomes,

$$J_{V,k}([Lg]_{V,k,SS}, [Lg]_{I,k,SS}) - L_k[Lg]_{I,k,SS} - k_{int,k}^{RLg}[RLg]_{I,k,SS}V_{I,k} = 0 \quad (24)$$

   Where

$$[Lg]_{V,k,SS} = R_{max,\,Lg,SS}$$

2) Cytokine-cytokine receptor complex in tissue interstitial space at steady-state is described by equation (25)

$$[RLg]_{I,k,SS} = \frac{[R]_{I,k,SS} \times [Lg]_{I,k,tot,SS}}{[R]_{I,k,SS} + K_D^{RLg}} \quad (25)$$

3) Derving free concentration of cytokine receptor in tissue interstitial space at steady-state

Let the free fraction of receptor at steady-state ($f_{R,k,SS}$) be defined as:

$$[R]_{I,k,SS} = f_{R,k,SS} \times [R]_{I,k,tot,SS} \quad (26)$$

Total cytokine receptor concentration in tissue interstitial space at steady-state is defined by equation (27)

$$[R]_{I,k,tot,SS} = [R]_{I,k,SS} + [RLg]_{I,k,SS} \quad (27)$$

Define

$$\alpha_k = \frac{[Lg]_{I,k,tot,SS}}{[R]_{I,k,tot,SS}}, \beta_k = \frac{K_D^{RLg}}{[R]_{I,k,tot,SS}}$$

Equation (27) can be transformed to equation (28)

$$1 = f_{R,k,SS} * (1 + \frac{\alpha_k}{f_{R,k,SS} + \beta_k}) \quad (28)$$

Once free fraction of cytokine receptor ($f_{R,k,SS}$) is solved using Newton interaction, free cytokine receptor concentration ($[R]_{I,k,SS}$) is calculated using equation (26), and free cytokine concentration at steady-state and synthesis rate of cytokine receptor at steady-state are computed using equations (29) and (30)

4) Free cytokine concentration in tissue interstitial space at steady-state is derived using equation (29):

$$[Lg]_{I,k,SS} = [Lg]_{I,k,tot,SS} - [RLg]_{I,k,SS} \quad (29)$$

5) Synthesis rate of cytokine receptor at steady-state is derived using equation (30)

$$k_{syn}^R = k_{deg}^R[R]_{I,k,tot,SS} - (k_{deg}^R - k_{int}^{RLg})[RLg]_{I,k,SS} \quad (30)$$

Results

Sensitivity Analysis

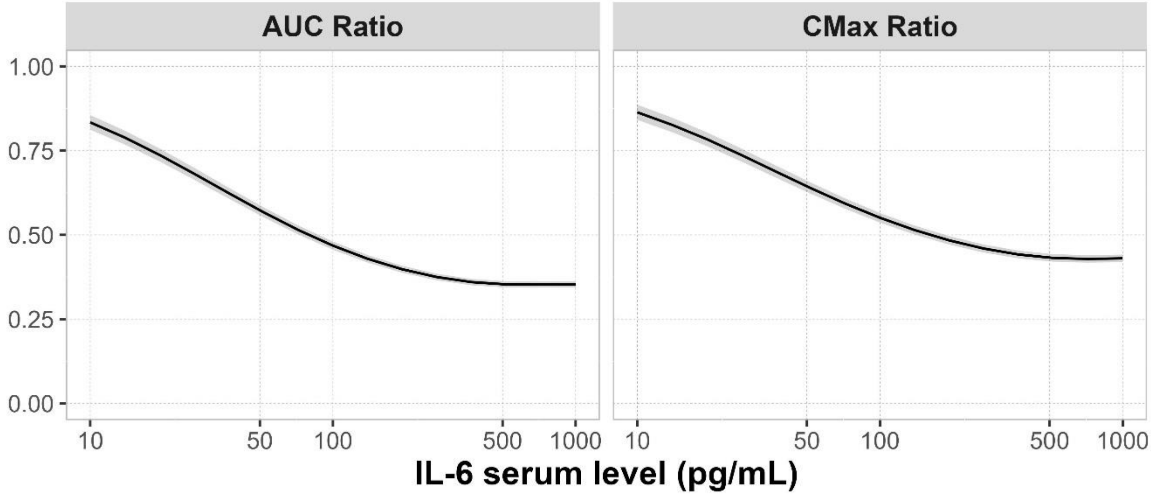

Figure S1. Population sensitivity analysis of baseline IL-6 concentrations (10–1000 pg/mL) on the AUC and C_{max} ratios of simvastatin following 1-week tocilizumab treatment. Simulations were performed in 10 trials of 12 virtual RA population (50% female, age range 28 to 72 years), aligned with clinical study [3]. Solid lines represent geometric mean, and shade areas represent 90% confidence interval of the total virtual population.

References:

1. Rippe, B.; Haraldsson, B. Fluid and protein fluxes across small and large pores in the microvasculature. Application of two-pore equations. *Acta Physiol Scand* **1987**, *131*, 411-428, doi:10.1111/j.1748-1716.1987.tb08257.x.
2. Aukland, K.; Reed, R.K. Interstitial-lymphatic mechanisms in the control of extracellular fluid volume. *Physiol Rev* **1993**, *73*, 1-78, doi:10.1152/physrev.1993.73.1.1.
3. Schmitt, C.; Kuhn, B.; Zhang, X.; Kivitz, A.J.; Grange, S. Disease-drug-drug interaction involving tocilizumab and simvastatin in patients with rheumatoid arthritis. *Clin Pharmacol Ther* **2011**, *89*, 735-740, doi:10.1038/clpt.2011.35.
